# A Joint De-Rain and De-Mist Network Based on the Atmospheric Scattering Model

**DOI:** 10.3390/jimaging9070129

**Published:** 2023-06-26

**Authors:** Linyun Gu, Huahu Xu, Xiaojin Ma

**Affiliations:** 1 School of Environmental and Chemical Engineering, Shanghai University, Shanghai 200444, China; 2 School of Computer Engineering and Science, Shanghai University, Shanghai 200444, China; huahuxu@shu.edu.cn; 3 Business School, Henan University of Science and Technology, Luoyang 471003, China; mxj@haust.edu.cn

**Keywords:** images de-rain, atmospheric scattering model, multi-scale convolution

## Abstract

Rain can have a detrimental effect on optical components, leading to the appearance of streaks and halos in images captured during rainy conditions. These visual distortions caused by rain and mist contribute significant noise information that can compromise image quality. In this paper, we propose a novel approach for simultaneously removing both streaks and halos from the image to produce clear results. First, based on the principle of atmospheric scattering, a rain and mist model is proposed to initially remove the streaks and halos from the image by reconstructing the image. The Deep Memory Block (DMB) selectively extracts the rain layer transfer spectrum and the mist layer transfer spectrum from the rainy image to separate these layers. Then, the Multi-scale Convolution Block (MCB) receives the reconstructed images and extracts both structural and detailed features to enhance the overall accuracy and robustness of the model. Ultimately, extensive results demonstrate that our proposed model JDDN (Joint De-rain and De-mist Network) outperforms current state-of-the-art deep learning methods on synthetic datasets as well as real-world datasets, with an average improvement of 0.29 dB on the heavy-rainy-image dataset.

## 1. Introduction

In reality, rainy days are a frequent occurrence that can significantly impact the accuracy of various computer vision systems, such as autonomous navigation and video surveillance [1,2,3], target detection and recognition [4,5,6,7]. Rain streaks formed by the refraction of raindrops and halos formed by the scattering of smaller raindrops (similar to mist, which will be referred to as mist later) in images can both restrict the use of video surveillance and autonomous driving target detection systems on rainy days. Therefore, the removal of rain effects from images or videos captured in rainy conditions and restoration of the background is a crucial task for computer vision systems.

In the past decade, video-based rain removal techniques have attracted more attention. Currently, the most mainstream approach is to use temporal information detection to remove rain streaks from videos. Rain removal from single images is a more challenging and widespread problem at this stage because adding temporal information will reduce the processing speed and cannot satisfy several scenarios with high real-time requirements, such as autonomous driving. This method of rain removal, which utilizes the temporality between frames, is not applicable to the more complicated task of removing rain from single images. Therefore, this paper focuses on the rain removal task of single images. In recent years, deep learning–based rain removal methods [8,9,10,11,12,13] have received much attention because of their powerful feature representation capabilities, and some methods have achieved good performance. Nevertheless, the deep learning methods are more likely to fall into overfitting due to the lack of guidance on physical principles, which leads to poor recovery results. Some scholars have attempted to use rainy-image reconstruction models that add rain-streak location information [9] and the effect of mist on atmospheric scene light [14]. However, these models also suffer from a poor ability to learn the detailed features of images.

To overcome these problems, this paper proposes a rain removal algorithm for a single image based on atmospheric scattering and multi-scale convolution. Firstly, we propose a method to construct rainy images based on the physical principle of atmospheric scattering. Our model considers the effects of both rain and mist layers on atmospheric scene light and background layer, unlike other models that only consider one or the other. To extract parameters in a rainy image, we designed a Deep Memory Block (DMB) for our proposed model. The DMB utilizes the Long Short-Term Memory (LSTM) network to selectively remember essential features for restoring rainy images. In addition, since rain and mist significantly differ in pixel size, we use the Multi-scale Convolution Block (MCB) in the model’s tail to obtain more contextual information. MCB supplements the model with more structural and detailed features by convolving kernels of different sizes in parallel.

The main contributions of this paper are summarized as follows:

1. The generation model of rainy images is reconstructed by defining rain streaks and mist as transmission media, and this model can initially remove the rain streaks and mist from the images. DMB is designed to selectively separate the rain layer and mist layer transmission spectrum in the rainy image, respectively.
2. MCB processes the reconstructed images to improve the robustness and accuracy of the model by learning more structural and detailed features which cannot be learned in the generative model.
3. Extensive experiments have proven that our design can produce more realistic results in the removal of rain streaks and halos, both qualitatively and quantitatively.

## 2. Related Work

There are two main approaches for rain removal from single images: models based on signal separation and models based on deep learning. This section briefly describes some related and competitive algorithms of these two approaches.

A. Rain removal models based on signal separation

Kang et al. [15] presented a rain removal method that divides the image input into the high-frequency part and low-frequency part, where the former represents the detail layer, including rain lines and object boundaries, while the later represents the structural layer of the image. This method separates the rain lines from the high-frequency part using sparse representation based on dictionary learning. The actual complexity of the network structure makes the generated images blurred at the edges due to the absence of high frequencies. Kim et al. [16] assumed that raindrops are elliptical vertical trajectories and used a nonlinear mean filtering method to eliminate rain lines from high-frequency messages. This technique is effective in some aspects but is poor at identifying rain streaks of other scales and shapes in their study. In their research, Luo et al. [17] identified rain lines in images with recognition capability using sparse coding, which could remove rain streaks while preserving image details better than in previous studies. However, the results are unsatisfactory, and the streaks remain visible in the images. Yu et al. [18] trained a Gaussian mixture model (GMM) from natural images to describe the prior knowledge of the background and rain layers. This approach can drastically reduce the amount of high-frequency information from the background layer to be removed as rain layer information. However, there are still some halos that cannot be removed entirely. These models based on signal separation focus on effectively distinguishing the high-frequency messages in the rain layer from the background. However, the current methods show poor results, and there are still relatively noticeable streaks and halos in the output images, which cannot be resolved completely at present.

B. Rain removal models based on deep learning

Deep learning has achieved great success in image rain removal in recent years. The methods based on deep learning describe rainy images as a superposition of background and rain layers. The rain layer accounts for all the noise in the image, including rain streaks and halos. Like [15], Yang et al. [8] also decomposed the rainy image into high and low-frequency parts, then mapped the high-frequency parts to the rain line layer for de-rain using a three-layer convolutional neural network. Yang et al. [9] believed that not only rain lines in heavy-rain images would affect the quality of the visual image, but also that the veiling effect resulting from the massive accumulation of rain lines would influence the images. Therefore, they proposed a novel pipeline: first detect the rain location, then estimate the rain line, and finally extract the clean background layer. A six-stage model was proposed by Ren et al. [10], where each stage takes the original rainy image and residuals from previous stages as inputs. Two models were classified according to whether LSTM was incorporated in each stage: PRN and PReNet, as well as PRNr and PReNetr with reduced parameters. Apart from those fully supervised methods mentioned above, there are also semi-supervised methods that have yielded good results. Wei et al. [11] proposed a semi-supervised method firstly to solve the rain removal problem by adding real-rain images and considering the residual between the synthetic rainy image and the corresponding ground-truth. The network can mitigate the problem of differences between real and synthetic data by adapting the supervised synthetic rain to the real unsupervised multiple rain types. Rajeev Yasarla et al. [12] modeled the potential space in the network using a Gaussian process, then generated a pseudo-GT image using a superposition of labeled data to approximate the unlabeled data, and finally used the pseudo-GT image to supervise the unlabeled data with the encoder.

Models based on deep learning commonly describe rainy images as a superposition of background and rain layers, and the majority of methods do not target haloes as a separate target noise for their removal. Compared to non-uniform rain streaks, halos are locally uniform, and removal methods that target rain streaks are not effective in removing halos. However, these halos will almost inevitably appear in every rainy image, especially in heavy-rain images. Therefore, if we do not consider its removal separately, heavy-rain images cannot be recovered well.

## 3. Methods

### 3.1. Network Architecture

Our rain removal model consists of two parts: a rain and mist model and a multi-scale convolution. The rain and mist model part is based on atmospheric scattering theory, where a single rainy image is used as input. Then, parameters are extracted from the rainy image, including the rain transfer spectrum, mist transfer spectrum, and atmospheric scene light transfer spectrum. The output of the first part is a preliminary clean image calculated from the extracted parameters based on the imaging principle formulation. In the rain and mist model, Deep Memory Block (DMB) and Atmospheric light Extraction Network (AEN) are included. DMB is used to separate the rain transfer spectrum and mist transfer spectrum from the input raw rainy image, and AEN is used to pre-train the atmospheric scene light transfer spectrum. The second part is the Multi-scale Convolution Block (MCB), which refines the estimated image from the first part and produces a cleaner image as the output.

We use a two-part stepwise removal of rain streaks in rain-containing images for the following reasons. The physical model based on atmospheric scattering is an abstraction of the real-rain image imaging process. The abstracted physical model provides constraints on the deep learning network to achieve better model feature learning capability, but the abstraction process also ignores many details. Therefore, in order to make our model more robust, we add a second part to it. MCB, which is a fully convolutional network without any physical model constraint, learns more features from the initially reconstructed image by several convolutions with varying kernel sizes. However, we cannot use this network alone either. MCB without physical model guidance has little effect on halo removal.

**Figure 1:**
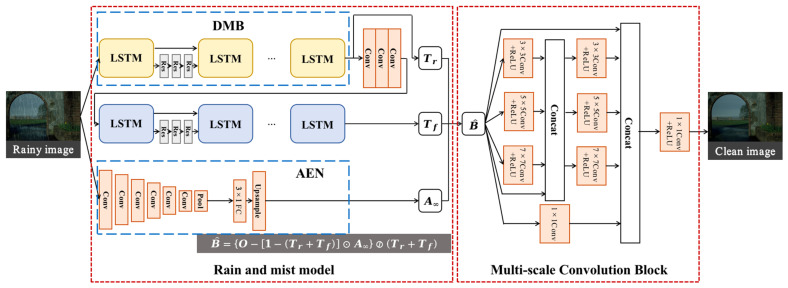
The general design network structure of Joint De-rain and De-mist Network (JDDN).

The network structure is shown in Figure 1. First, the raw rainy image initially passes through the rain and mist module to extract the rain transfer spectrum, mist transfer spectrum and atmospheric scene light transfer spectrum. The original image is passed through the DMB to extract the rain transfer spectrum. Then, after three layers of convolution, the mist transfer spectrum is extracted by DMB. DMB is a modified Long Short-Term Memory (LSTM) network, which selectively forgets the unimportant features of the previous node and remembers the important ones, amplifying the constraint effect of the physical model on the rain and mist feature extraction. After the DMB, the rain transfer spectrum is filtered out from the original rainy image. Then, using a three-layer convolution, the mist transfer spectrum is filtered out from the rain transfer spectrum. The reason for this operation is that the features extracted after three-layer convolution are more homogeneous, which is more consistent with the characteristics of the mist layer. The atmospheric scene light is obtained by an encoded CNN network, AEN. The original rainy image is pre-trained by AEN to obtain a relatively accurate estimate of the atmospheric light. After obtaining the transfer spectrum variables and the atmospheric scene light variables in the rain and mist model, preliminary rain-removal results are obtained according to our proposed rain and mist model formulation. Subsequently, the preliminary results are fed to MCB, which learns more features at different levels by multi-scale convolution with residual connections. The convolutions of different sizes learn as many image detail features as possible while stabilizing the structural features, and the rich features help the network to obtain cleaner output images.

### 3.2. Rain and Mist Model Based on Atmospheric Scattering

The scattering model was first used in the field of fog removal of images. When taking pictures on a foggy day, the light source received by the sensor is disturbed by the fog. The collected light mainly comes from the reflected light of the target after particle attenuation and the atmospheric light formed by scattering. Thus, the fog-containing image I(x) based on the atmospheric scattering model can be expressed as:

(1) $$I(x)=D+A=J(x)t(x)+A_{\infty }(1-t(x))$$

where D denotes the reflected light of the target after particle attenuation, A denotes the atmospheric light formed by particle scattering, t(x) is the atmospheric projectivity, J(x) denotes the reflected light of the target, J(x)t(x) denotes the reflected light of the target after attenuation D, $A_{\infty }$ denotes the original light intensity from the light source to the sensor, and $A_{\infty }(1-t(x))$ is the light intensity after scattering A.

The idea of recovering clean images from foggy images is to estimate the transfer function t(x) and atmospheric light $A_{\infty }$ from foggy images based on various a priori knowledge or tools of image processing, and to recover clean images by substituting the solved parameters into the atmospheric scattering model.

Since the halo in the rainy image is also obtained due to the scattering of fog-like small droplets, we consider the removal of the halo in the rainy image using the scattering model and describe the imaging process for the rainy image O as follows:

(2) $$O=\left(T_{r}+T_{f}\right)⊙B+\left[1-\left(T_{r}+T_{f}\right)\right]⊙A_{\infty }$$

where O denotes rainy images, B denotes clean background images, and $A_{\infty }$ denotes atmospheric light before attenuation. $T_{r}$ and $T_{f}$ denote the transmission spectrum of the rain layer and the mist layer, respectively. In addition, 1 in Equation (2) stands for All-one Matrix, and ⊙ is used for the pixel-by-pixel multiplication operation. We consider that the actual rain scene, the rain layer and the mist layer both affect the reflection of the background image and cause the atmospheric light attenuation, so we treat the rain layer and the mist layer as a whole. However, because they are different in the image, the rain layer usually looks thin and long, while the mist layer covers a larger area, and it will cover the image more evenly. Therefore, we divide the transfer medium into the rain layer transfer spectrum and mist layer transfer spectrum and extract their features separately. The proposed model extends all variables to the same dimension, and therefore the relationships between the variables are expressed using a pixel-by-pixel product.

For the mathematical Equation (2), if we want to obtain the result of removing the rain B, the inverse solution will be obtained as follows:

(3) $$\hat{B}=\left\{O-\left[1-\left(T_{r}+T_{f}\right)\right]⊙A_{\infty }\right\}⊘\left(T_{r}+T_{f}\right)$$

where ⊘ denotes a pixel-by-pixel division.

According to Equation (3), if we want to obtain clean images $\hat{B}$ after preliminary rain removal, we need to predict the rain layer transfer spectrum $T_{r}$ and mist layer transfer spectrum $T_{f}$ as well as the scene atmospheric light $A_{\infty }$, respectively. In the following, we introduce the Deep Memory Block (DMB) for predicting the rain layer transfer spectrum $T_{r}$ and the mist layer transfer spectrum $T_{f}$, and the Atmospheric Light Extraction Network (AEN) for predicting the scene atmospheric light $A_{\infty }$.

### 3.3. Deep Memory Block (DMB)

**Figure 2:**
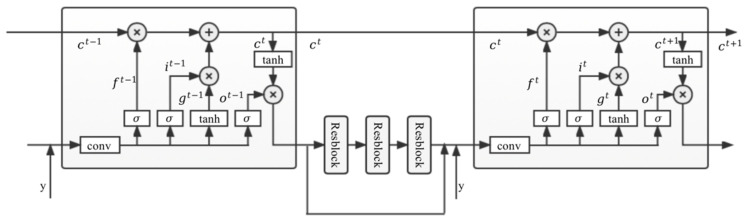
Schematic diagram of the Deep Memory Block (DMB).

In order to predict the rain layer transfer spectrum $T_{r}$ and the mist layer transfer $T_{f}$ spectrum separately, we propose a DMB that selectively learns the rain layer and mist layer features using an improved LSTM in a recursive manner. The LSTM module includes forgetting gates, input gates and output gates, where the forgetting gates are mainly used to selectively forget some unimportant features from the previous stage. We choose the LSTM for learning features for transfer spectrum prediction because it can filter out a large amount of unimportant contextual information and ensure that the learned features can be better adapted to our proposed scattering-based model. Figure 2 shows the schematic structure of the DMB prediction module phase t−1 and t:

DMB uses a design with 6 LSTM blocks connected to each other and 3 ResBlock blocks between each of 2 LSTM blocks. Each stage of the image passes through 1 LSTM and 3 ResBlock modules, and their output results are fused with the target image and sent to the next stage of the network. In this way, a more realistic rain and mist layer transfer spectrum can be obtained in each stage of extraction. Firstly, the extraction process of rain layer transfer spectrum $T_{r}$ is introduced, and the intermediate stages of rain layer transfer spectrum $r^{t}$ for stage t are:

(4) $$r^{t}=F_{r}\left(y,r^{t-1}\right)$$

where, $r^{t}$ denotes the transfer spectrum of the output of the stage t, y denotes the target ground-truth, $r^{t-1}$ denotes the transfer spectrum of the output of the stage t−1, and $F_{r}$ denotes the algorithm of the first stage of the DMB.

The 4 components in Equation (4) are described below, where all convolution kernels are 3 × 3, padding is 1, and step size is 1.

(1) Input layer $f_{in}$: The output from the previous stage $r^{t-1}$ and the target image y are stitched together, and they are fused by one layer of convolution and ReLU. The input in this layer, including the output of the previous stage $r^{t-1}$ and the target image y, has 3 channels separately and the number of output $z^{t}$ channels is 32. The output $z^{t}$ of the input layer $f_{in}$ can be expressed as:

(5) $$z^{t}=f_{in}\left(y,r^{t-1}\right)$$

(2) Recurrent layer $f_{recurrent}$: In the stage t, the input of $f_{recurrent}$ is the result of the output of the input layer $z^{t}$ and the transfer spectrum $h^{t-1}$ in the previous stage of the recurrent layer. They are fed into the LSTM block as variables to get the new transfer spectrum $h^{t}$. This process can be expressed as follows:

(6) $$h^{t}=f_{recurrent}\left(z^{t},h^{t-1}\right)$$

LSTM or GRU can be used here for $f_{recurrent}$. According to [19,20], LSTM can have a better boosting effect in the experiment, so LSTM is chosen in this model. For LSTM, it should include input gate $i^{t}$, forgetting gate $f^{t}$, output gate $o^{t}$ and cell state $c^{t}$. The formula is as follows:

(7) $$i^{t}=\sigma \left(W_{iz}⊗z^{t}+W_{is}⊗h^{t-1}+b_{i}\right)\;f^{t}=\sigma \left(W_{fz}⊗z^{t}+W_{fs}⊗h^{t-1}+b_{f}\right)\;o^{t}=\sigma \left(W_{oz}⊗z^{t}+W_{os}⊗h^{t-1}+b_{o}\right)\;g^{t}=tanh\left(W_{gz}⊗z^{t}+W_{gs}⊗h^{t-1}+b_{g}\right)\;c^{t}=f^{t}⊙c^{t-1}+i^{t}⊙g^{t}\;h^{t}=o^{t}⊙tanh\left(c^{t}\right)$$

where ⊗ denotes 2D convolution; ⊙ is the Hadamard product, which is the multiplication of the corresponding elements in the operation matrix; σ is the sigmoid function; W and b is the corresponding convolution matrix parameters and bias vector. There are 32 channels of convolutional inputs and outputs in all LSTMs.

(3) Residual layer $f_{res}$: $f_{res}$ is the key part of the rain removal model for extracting depth features. In this part, the depth features of $h^{t}$ are extracted by 3 ResBlocks, each of which includes 2 convolutional layers and ReLU [21]. All convolutional layers receive 32 channels of features without adding upsampling or downsampling operations.

(4) Output layer $f_{out}$: The features of 32 channels obtained in step 3 $f_{res}\left(h^{t}\right)$ are fused with the transfer spectrum $h^{t}$ obtained in step 2 through a convolution for feature fusion as the output $r^{t}$ containing RGB 3 channels. This process can be expressed as follows:

(8) $$r^{t}=f_{out}\left(f_{res}\left(h^{t}\right),h^{t}\right)$$

The output after six stages of $r^{t}$ is the rain layer transfer spectrum, $T_{r}$. According to Wang Y.L. et al. [22], the mist layer transfer spectrum can be further extracted from the rain layer transfer spectrum, and adding three sets of convolutions after the rain layer transfer spectrum can make the obtained feature map more homogeneous, which is exactly in line with the characteristics of the mist layer transfer spectrum. We also adopt this design and send the output rain transfer spectrum $T_{r}$ to the DMB module after the convolution operation to extract the mist transfer spectrum $T_{f}$.

### 3.4. Atmospheric Light Extraction Network (AEN)

To predict the scene atmospheric light $A_{\infty }$, we designed an Atmospheric light Extraction Network (AEN). The structure of the AEN is shown in Figure 1. Since the atmospheric light is often considered as a constant in the whole rain scene, the output of AEN is a vector of 3 × 1. To facilitate the calculation of the rain and mist model, this vector is expanded to the same dimension as the image with RGB 3 channels. For a given pixel, when $T_{r}+T_{f}=0$, according to Equation (2) $O=A_{\infty }$ is obtained. Thus, it can be seen that, under optimal conditions, the atmospheric scene light is numerically equal to the value of this pixel. Further, we just need to find the rain pixel that is subject to the smallest rain and mist layer transfer spectrum of the rain-containing image to use it instead of the scene atmospheric light. Yang W et al. [9] found a method to locate the rain pixel. We first find the atmospheric light by pre-training it, and then incorporate AEN into the whole network for joint training to get the most suitable atmospheric light $A_{\infty }$. We could get $A_{\infty }$ by minimizing the loss, and the loss function equation is as follows.

(9) $$L_{A}=\frac{1}{N}\sum_{n=1}^{N}{\left\|A\left(I_{n}\right)-A_{n}\right\|}^{2}$$

where $A\left(I_{n}\right)$ denotes the scene atmospheric light $A_{\infty }$ obtained by the AEN, $A_{n}$ denotes the scene atmospheric light $A_{\infty }$ obtained by pre-training, and N denotes the number of training samples.

### 3.5. Multi-Scale Convolution Block (MCB)

**Figure 3:**
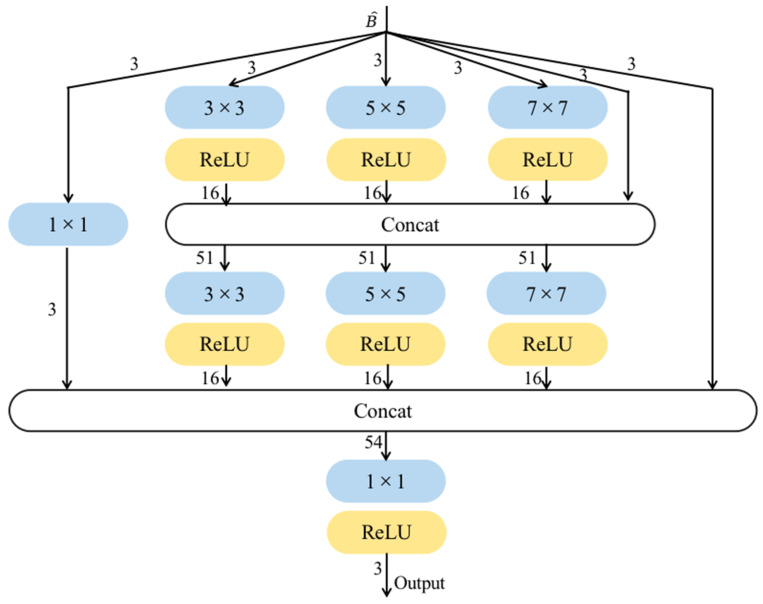
Structure of Multi-scale Convolution Block (MCB).

The second part of our model is MCB, which is not limited by the physical model to learn features, and it is not only able to learn image structure features but also has a better learning effect on detailed features that are not easily expressed by the physical model. Figure 3 depicts the detailed structure of MCB. The number marked on the arrow in the blue box indicates the number of input channels for convolution, and the number on the arrow from the yellow box indicates the number of output channels for convolution. Each stitching operation involves 4 or 5 feature mappings that enable the learning of local features in the image. One of the mappings comes directly from the output of the previous layer, while the others are obtained through convolutional kernels of different sizes used to detect features at different scales. Small-scale convolutional kernels are used to learn the image detail features better, while the large-scale convolutional kernels are used to stabilize the image structure. Finally, they are connected into a 1 × 1 convolution layer for feature fusion to improve computational efficiency.

“Concat” represents the collocation operation. The numbers in the blue box represent the size of the convolution kernel, and the yellow box represents the use of the ReLU function (Linear Rectification function) as the activation function. In order to make the splicing operation easier, each layer of the MCB uses a convolution kernel with a stride of 1. The final 1 × 1 convolutional layer in the MCB reduces the feature map to match the number of input channels, ensuring that both input and output of the block have identical number of channels.

These operations are represented as follows:

(10) $$Y_{1}=L\left(\omega_{1\times 1}^{1}*\hat{B}\right)$$

(11) $$S_{1}=L\left(\omega_{3\times 3}^{1}*\hat{B}\right)$$

(12) $$W_{1}=L\left(\omega_{5\times 5}^{1}*\hat{B}\right)$$

(13) $$Q_{1}=L\left(\omega_{7\times 7}^{1}*\hat{B}\right)$$

(14) $$S_{2}=L\left(\omega_{3\times 3}^{2}*concat\left[S_{1},W_{1},Q_{1},\hat{B}\right]\right)$$

(15) $$W_{2}=L\left(\omega_{5\times 5}^{2}*concat\left[S_{1},W_{1},Q_{1},\hat{B}\right]\right)$$

(16) $$Q_{2}=L\left(\omega_{7\times 7}^{2}*concat\left[S_{1},W_{1},Q_{1},\hat{B}\right]\right)$$

(17) $$X_{n}=L\left(\omega_{1\times 1}^{3}*concat\left[{Y_{1},S}_{2},W_{2},Q_{2},\hat{B}\right]\right)$$

where ω denotes the weight, and in order to simplify notations to ignore the bias, * represents the convolution calculation. The superscript shows the location of the convolution, whereas the subscript means the scale of the kernel. $L\left(\cdot ,\cdot \right)$ denotes the activation function ReLU.

More details of the algorithm are given in Algorithm 1.
**Algorithm 1.** Obtain the optimal parameters of the model**Input:** The output of rain and mist model $\left\{\hat{B_{r}^{\left\{t\right\}}}\right\}$ 1: for *i* = 1 to epoch do: 2:  for *j* = 1 to batchnum do: 3:   Convolution of $\left\{\hat{B_{r}^{\left\{t\right\}}}\right\}$ by Equations (10)–(13) to get $\left\{Y_{1}^{\left\{t\right\}}\right\}$, $\left\{S_{1}^{\left\{t\right\}}\right\}$, $\left\{W_{1}^{\left\{t\right\}}\right\}$, $\left\{Q_{1}^{\left\{t\right\}}\right\}$. 4:    Concat $\left\{S_{1}^{\left\{t\right\}}\right\}$, $\left\{W_{1}^{\left\{t\right\}}\right\}$, $\left\{Q_{1}^{\left\{t\right\}}\right\}$ with $\left\{\hat{B_{r}^{\left\{t\right\}}}\right\}$ to get $\left\{C_{1}^{\left\{t\right\}}\right\}$. 5:    Convolve $\left\{C_{1}^{\left\{t\right\}}\right\}$ with Equations (14)–(16) to get $\left\{S_{2}^{\left\{t\right\}}\right\}$, $\left\{W_{2}^{\left\{t\right\}}\right\}$, $\left\{Q_{2}^{\left\{t\right\}}\right\}$. 6:    $\left\{O_{1}^{\left\{t\right\}}\right\}$, $\left\{S_{2}^{\left\{t\right\}}\right\}$, $\left\{W_{2}^{\left\{t\right\}}\right\}$, $\left\{Q_{2}^{\left\{t\right\}}\right\}$ and $\left\{\hat{B_{r}^{\left\{t\right\}}}\right\}$ are convolved by Equation (17) to obtain $\left\{\hat{X_{r}^{\left\{t\right\}}}\right\}$. 7:    Update the intermediate parameters by loss functions Equations (18)–(19) 8:  end for 9. end for **Output:** The learned optimal parameters.

### 3.6. Loss Function

In the case of a training JDDN with T stages, there are T outputs, i.e., $x^{1},x^{2},\cdots ,x^{T}$ and it is quite natural to apply recursive supervision to each intermediate result.

(18) $$L=\sum_{t=1}^{T}\lambda_{t}l\left(x^{t},x^{gt}\right)$$

where $x^{gt}$ is the corresponding ground-truth, $l\left(\cdot ,\cdot \right)$ indicates the variance between the output of JDDN at stage t and the corresponding ground-truth, and $\lambda_{t}$ means a trade parameter.

As for the choice of $l\left(\cdot ,\cdot \right)$, negative SSIM loss [23], MSE loss, MSE + SSIM [24], +SSIM [25], or adversarial loss [26], have been widely used to train denoising networks. Negative SSIM loss works better than MSE loss and those hybrid loss functions quantitatively and qualitatively [27] for the recurrent network architecture. As a result, the negative SSIM loss is chosen as the loss function. For a rainy image a and its corresponding ground-truth $a^{gt}$, $l\left(\cdot ,\cdot \right)$ can be expressed as:

(19) $$l\left(a,a^{gt}\right)=-SSIM\left(a,a^{gt}\right)$$

## 4. Experiments

In this section, the image recovery capability of JDDN is experimentally verified. First, we qualitatively analyze the visual effects of the rainy images by comparing different models on the rain-removal output images visually, and quantitatively by calculating the peak signal-to-noise ratio (PSNR) and structural similarity ratio (SSIM) of the generated images. Both PSNR and SSIM are larger to indicate better image recovery quality. Then, the generalization performance of the model is verified on real-world rain images.

### 4.1. Dataset

All the training and testing sets used to train the JDDN model are listed in Table 1.

In our experiments, three training sets are used for training and seven testing sets are used for testing. Among them, the SPA-data testing set is the real-world rain image testing set, and the other training and testing sets are synthetic datasets where rainy images are generated by overlaying rain streak on the ground-truth images. All of these datasets mentioned above are publicly available.

The training set RainTrainH is the Rain_heavy_train training set provided in [10] after removing 546 pairs of duplicate image sets, and it contains 1254 pairs of heavy-rain images and ground-truth. RainTrainL is the Rain_light_train light-rain training set in [10] and includes 200 pairs of light-rain images and ground-truths. Rain12600 includes 900 sets of the training set provided in [28], each of which includes a ground-truth and 14 rainy images containing different types of synthesized rain streaks.

The testing sets Rain100H and Rain200H are the heavy-rain image testing sets provided by [10], which contain 100 and 200 pairs of synthetic heavy-rain images and ground-truths, respectively. The former is the dataset at the time of the paper’s publication and the latter is the subsequently updated one. Rain100L, Rain200L are also testing sets provided by [10], but they are light-rain testing sets. They contain 100 and 200 pairs of rainy images with their corresponding ground-truths, respectively. Regardless of the testing set of heavy-rain images or the testing set of light-rain images, the updated testing set has no inclusion relationship with the initial testing set. Rain12 is the testing set provided by [18], which includes 12 pairs of synthetic rainy images and their corresponding ground-truths. The rain streaks superimposed in Rain12 are more irregular and more similar to the real-rain images. Rain1400 is a testing set of 100 images provided by [28], each including a ground-truth and 14 synthesized rainy images with different types of rain streaks. SPA-data is a dataset of 1000 real-rain images provided by [29]. The ground-truth is also provided in [29] for subsequent researchers to perform quantitative analysis of the results. These ground-truths are calculated by the authors from the temporal differences of the videos with rain. Therefore, the ground-truths of the SPA-data dataset are not fully truthful and the quantitative analysis results serve as a reference only.

In the subsequent experiments, the testing sets Rain100H, Rain200H, Rain100L, Rain200L, and Rain12 are used to verify the model’s performance to remove a single type of rain streak. Among them, the testing sets Rain100H, Rain200H, and Rain12 are used to validate the performance of the model on heavy-rain images, and the testing sets Rain100L and Rain200L are used to validate the performance of the model on light-rain images. The testing set Rain1400 is used to verify the model’s ability to recover images containing multiple types of rain streaks. The testing set SPA-data is used to test the robustness of the model for real-world rain images.

### 4.2. Training Details

Our model is implemented using Pytorch and trained on a PC equipped with NVIDIA RTX 3060 GPU. The models use the same training settings except for specific statements. Patch size is 100 × 100, and batch size is 12. In addition, the learning rate is 1 × 10^−3^, ending after 250 epochs.

### 4.3. Recovery of Heavy Rain Images with Single Rain Streaks

In this section, the RainTrainH training set will be used for training, and the rain-removal effect of the model on a single heavy-rain image will be evaluated by the performance of the model on the Rain100H, Rain200H and Rain12 testing sets.

**Figure 4:**
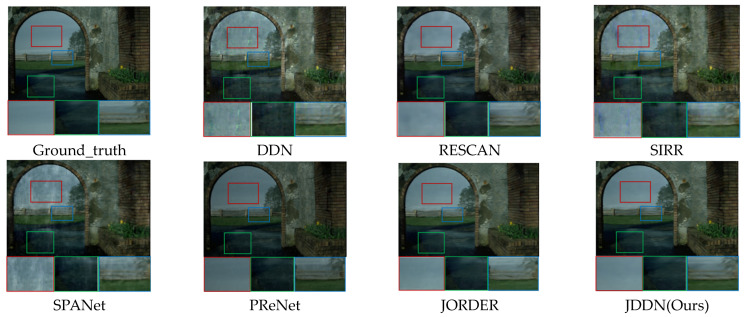
Comparison of the visual quality of the JDDN model on synthetic rainy-image dataset Rain200H.

**Qualitative analysis.** The rain-removal results of several advanced rain removal models on Rain200H are shown in Figure 4, including DDN [28], RESCAN [29], SIRR [11], SPANet [30], PReNet [10], and JORDER [9]. The research of DDN, RESCAN, SIRR, and SPANet focuses only on how to extract rain streaks, so their results only remove the rain streaks, and the halos are still present. Although PReNet does not intentionally focus on the removal of halos, it uses a shallow six-stage ResNet to identify the noise in the rainy images more accurately, and both rain streaks and halos are learned. However, as can be seen from the images marked by the red boxes, the brightness of the images is not fully recovered. Similar to our JDDN model, JORDER uses a stepwise method of rain and fog layer removal, which is more effective in removing the rain and mist layers of the images. Besides, due to the detailed feature extraction of the multi-scale convolution block in the second part of our model, our model is superior to JORDER in some details, as shown in the green box and blue box. Our model JDDN is successful in recovering both color and detail recovery.

**Quantitative analysis.** To verify the superiority of JDDN, we compared it with other state-of-the-art models. The models we compared include DDN, RESCAN, SIRR, SPANet, PReNet, JORDER, RFCTNet [31], GN [32]. The data were used with published codes for the same training and testing sets to obtain fair evaluation results. The specific quantitative analysis results are shown in Table 2. Two evaluation metrics, PSNR and SSIM, are used for the quantitative analysis, and both metrics have larger values to indicate better image recovery, and the metric with the best results is indicated in bold. Compared with other advanced models, including SIRR with semi-supervised strategy and RFCTNet and GN with self-attentive mechanism, JDDN ranked first in both PSNR and SSIM on the two heavy-rain datasets Rain100H and Rain200H as well as the irregular rain-streak dataset Rain12, indicating that JDDN has significantly improved the recovery of heavy-rain images after adding the special design for halo.

### 4.4. Recovery of Light Rain Images with Single Rain Streaks

In this section, the RainTrainL training set will be used for training, and the rain removal effect will be evaluated by analyzing the performance of the model on the Rain100L and Rain200L testing sets.

**Figure 5:**
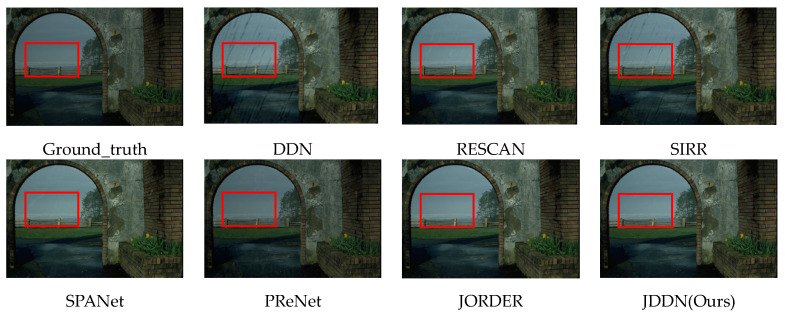
Comparison of visual quality of JDDN model on synthetic rainy-image dataset Rain200L.

**Qualitative analysis.** The rain-removal results of several advanced rain removal models on Rain200L are shown in Figure 5. In this section, the same models as in the previous section are still used for comparison of the rain removal performance. From Figure 5, it can be seen that DDN and SIRR, which only consider rain-streak removal, still have unsatisfactory rain-removal results in light-rain images. RESCAN and SPANet achieve better rain-removal results than DDN and SIRR after special network design, but there are still a small number of rain streaks and halos remaining, as shown in the red box. In the recovery results of the light-rain images, both JDDN and JORDER achieved good results. Comparing Figure 4 with Figure 5, we can intuitively see that most of the models will have significantly better removal results for the light-rain images than the heavy-rain images. This is because the interlaced rain streaks in the heavy-rain images will show more mist-like masking effects, and if we simply consider only the removal of rain streaks, the halo in the heavy-rain images will not be removed, while this problem almost does not exist in the light-rain images. So whether or not a de-mist design is added, good results can still be achieved.

**Quantitative analysis.** Table 3 shows the comparison of the average PSNR and SSIM of different algorithms on the Rain100L and Rain200L testing sets, with the bold text representing the best performance. DDN and SIRR with unrecoverable images perform the worst. RESCAN and PReNet have significant differences in metrics for different testing sets, indicating their poor generalization performance. SPANet, JORDER, RFCTNet, GN and JDDN achieve good results on both testing sets. Since the light-rain images are less affected by halos, our model does not have a significant advantage over other advanced models, but it also has top removal and generalization capabilities.

### 4.5. Recovery of Images Containing Multiple Types of Rain Streaks

To verify the generalization ability and robustness of JDDN for various types of rain streaks, we tested on another dataset, Rain1400. Rain100H, Rain100L and other testing sets are synthesized for the single type of rain streaks on every clean image, while the Rain1400 testing set is synthesized for 14 types of rain streaks on every clean image. This set of experiments was trained on the Rain12600 training set.

**Figure 6:**
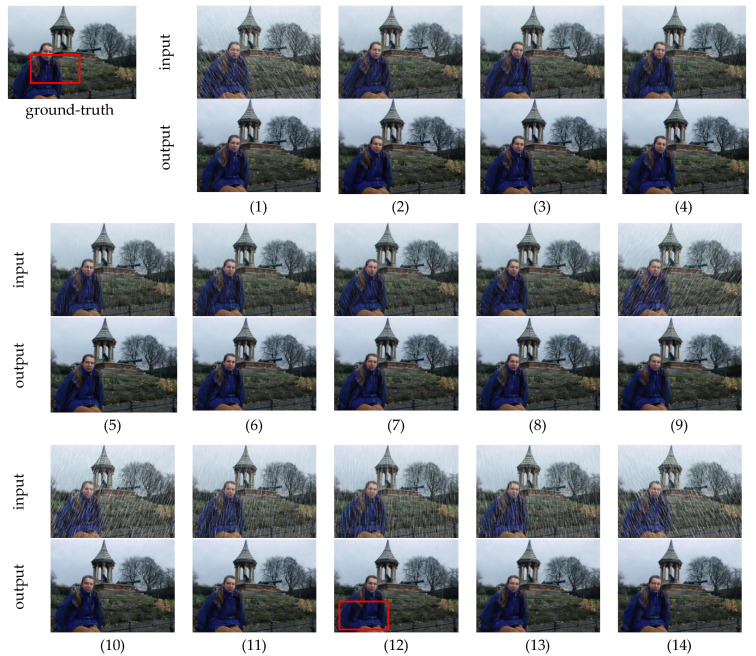
The results of JDDN on Rain1400 dataset.

**Figure 7:**
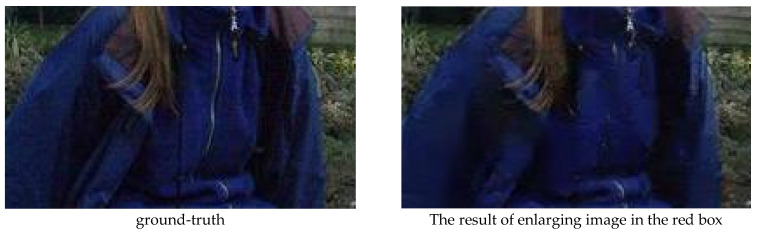
Enlarged details of JDDN’s test results on the Rain1400 dataset.

**Qualitative analysis.** The test results of JDDN on the Rain1400 dataset are shown in Figure 6. The first image in Figure 6 is the ground-truth, and the remaining images are 14 sets of rainy images containing different types of rain streaks and their corresponding output results. It can be seen that for these 14 synthetic rain streaks, JDDN almost achieves the goal of rain-streak removal. Compared with the large rain images (1, 9–14), the processing results of the small rain images (2–8) are more excellent, and there are still some details that cannot be fully recovered in the large rain images, as shown in Figure 7. The two images shown in Figure 7 are the images after enlarging the details in the red box of Figure 6. Like the results of the previous section for the heavy-rain image and the light-rain image, the recovery of the light-rain image by JDDN is better than that of the heavy-rain image.

**Quantitative analysis.** The comparison results of JDDN on the Rain1400 dataset are shown in Table 4. DDN provides the model for [25], which is the same as the Rain1400 dataset. The results in Table 4 indicate that JDDN outperforms both DDN and SIRR.

### 4.6. Recovery of Real Rain Images

This section describes the rain removal effect of JDDN on a real-rain image dataset. Since real-world rain streaks are more diverse in shape, direction, and intensity, we use Rain12600 as the training set and SPA-data as the testing set. In recent years, many models on synthetic datasets have made great progress and breakthroughs in the field of image rain removal, but they are limited by the fact that synthetic datasets cannot simulate rain streaks realistically to an extent, which makes it more difficult for these past models to process real-rain images.

Due to the unique characteristics of real images, it is impossible to capture rainy images and their corresponding clean images simultaneously. For a long time, there has been no publicly available benchmark for quantitative comparison of real-rain images, and evaluation models can only be compared qualitatively based on their effectiveness in removing rain from such images. Wang et al. [26] used a semi-automatic method that fuses temporal prior and manual supervision to generate high-quality clean images from each input sequence of real images. Using this method, a massive dataset of SPA-data consisting of 29.5 k rainy images and clean images was created, covering a wide range of natural rainy scenes. This section adopts this dataset as a real-image dataset for evaluating the performance of JDDN in real-world generalization and compares its output results qualitatively and quantitatively (PSNR and SSIM).

**Figure 8:**
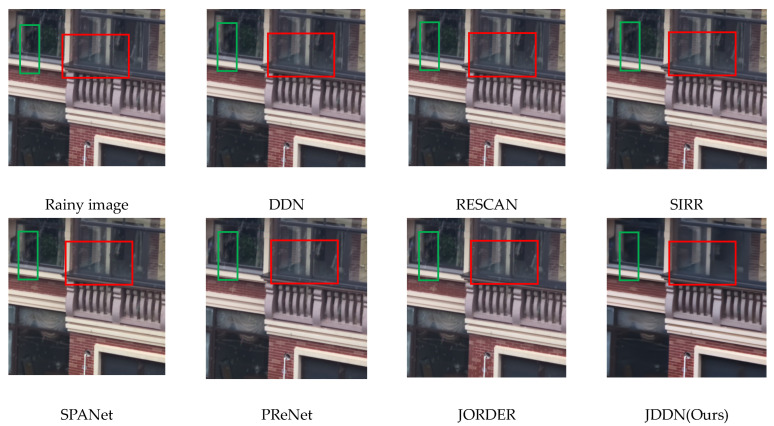
Visual comparison of processing results for real-rain images on the SPA-data dataset.

**Qualitative comparison.** A comparison of the results of JDDN with other models on the real-rain image dataset is shown in Figure 8. Compared with other methods, JDDN has a clear advantage in rain-streak removal. Most of the rain streaks are removed, but they are still a few residual rain streaks in the denser dark background. As shown in the red box, only the rain streaks in JDDN are removed cleanly. DDN, RESCAN, and SIRR can recover better for dark window frames, while SPANet, PReNet, JORDER, and JDDN make the image smoother while removing rain streaks, as shown in the green box. For recovering items with similar background colors, such smoothing is detrimental to image recovery. Further improvements need to be made in this regard.

**Quantitative comparison.** Table 5 shows the test results obtained by various different models on the testing set of SPA-data. It can be seen that there are no significant differences in the values of PSNR and SSIM for each model, and JDDN also gets good results. However, some models that intuitively feel that rain streaks cannot be removed cleanly also end up with better values. The reason is that the clean images in this dataset are derived from the images extracted from the rainy-image sequences. Although their effect is close to the real one and is currently recognized as the best real dataset, they are still the result obtained through the processed rainy images. Therefore, the quantitative comparison values are only for reference.

### 4.7. Ablation Experiments

In order to verify the necessity of each module design of the JDDN model, we conducted ablation experiments. The verification is divided into three parts: (1) using only the Rain and Mist Model RMM; (2) using only the Multi-scale Convolution Block MCB; (3) no LSTM in the rain and mist model and using ordinary convolution instead of LSTM for learning rain and mist transfer spectrum features (NLSTM). We trained these three parts separately and finally tested them in Rain100H, Rain100L, Rain200H, Rain200H, and Rain12. The results of PSNR and SSIM are shown in Table 6. The combination of RMM with MCB significantly optimizes rain-removal results. In addition, LSTM greatly improves the model’s performance. When a single convolution is used, the predicted rain and mist transfer spectrum is very unsatisfactory.

**Figure 9:**
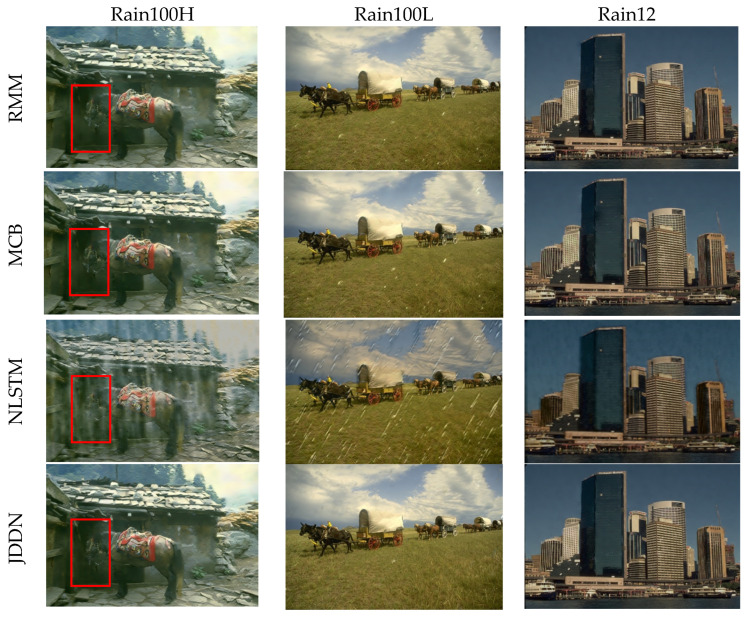
Visual comparison of the effect of removing the different modules.

Figure 9 shows the output images of the three-part ablation experiment. It can be seen that the model without LSTM is unsatisfactory in terms of both the rain-streak removal and image color recovery, as it hardly recovers the original image. RMM and MCB generally have an effect on rain-streak removal. However, there are still some white spots on the grass for light-rain images. For heavy-rain images, rain streaks can almost be removed, but the recovery of color and details in the image is slightly inferior to our JDDN. For example, there are still some white streaks residues in the red box. The combination of RMM and the MCB allows the model to learn more structural and detailed features, which leads to good results in both rain-streak removal and halo removal, thus improving image quality.

## 5. Discussion

In this paper, a new rain and mist model based on the atmospheric scattering model and single-image rain removal with a multi-scale convolution block (JDDN) is proposed, which can have a good effect on removing rain streaks as well as haloes caused by atmospheric light scattering in the image. First, we propose a new rain image synthesis process based on the atmospheric scattering model in the rain and mist description model to reconstruct the rain image, and get the preliminary clean image according to the inverse process of this model, wherein we use the DMB to predict the rain and mist layer transfer spectra in the model; the LSTM structure is used in the block to selectively memorize those features that are more adapted to our proposed model. In addition, JDDN uses MCB to learn more structural and detailed features of the initially processed images without physical model constraints, to improve the subjective and objective evaluation of rain-removal images.

Although satisfactory results are achieved in various classes of rainy images, JDDN still has some limitations. For example, the recovery of some fine items with similar color to the background in real-rain images still needs improvement. In the future, we can consider incorporating real-rainy-day images into the training model and try some semi-supervised or unsupervised models to obtain better rain-removal results for real rainy-day images.

## Figures and Tables

**Table 1:** Datasets used for experimental training and testing.

	Training Sets	Testing Sets
Datasets of heavy-rain images	RainTrainH [10]	Rain100H [10]
	RainTrainH [10]	Rain200H [10]
Datasets of light-rain images	RainTrainL [10]	Rain100L [10]
	RainTrainL [10]	Rain200L [10]
Datasets of irregular rain-streak images	RainTrainH [10]	Rain12 [18]
Datasets of multiple types of rain streaks	Rain12600 [28]	Rain1400 [28]
Datasets of real-rain images	Rain12600 [28]	SPA-data [29]

**Table 2:** Comparison of the average PSNR and SSIM of different algorithms on the heavy-rain dataset (PSNR/SSIM). Bolded texts represent the best performance.

	DDN	RESCAN	SIRR	SPANet	PReNet	JORDER	RFCTNet	GN	JDDN (Ours)
Rain100H	21.92/ 0.764	28.64/ 0.864	22.47/ 0.716	26.54/ 0.843	29.83/ 0.899	26.54/ 0.835	29.02/ 0.900	29.48/ 0.876	**29.79/** **0.907**
Rain200H	22.03/ 0.713	28.02/ 0.862	22.17/ 0.719	26.59/ 0.869	29.36/ 0.903	29.21/ 0.891	29.12/ 0.903	29.37/ 0.905	**29.43/** **0.908**
Rain12	31.78/ 0.900	-	34.02/ 0.935	32.02/ 0.855	34.45/ 0.938	33.92/ 0.953	35.50/ 0.969	34.78/ 0.957	**35.99/** **0.969**

**Table 3:** Comparison of mean PSNR and SSIM of different algorithms on the light-rain dataset. Bolded texts represent the best performance.

	DDN	RESCAN	SIRR	SPANet	PReNet	JORDER	RFCTNet	GN	JDDN (Ours)
Rain100L	32.16/ 0.936	29.80/ 0.881	32.37/ 0.926	35.66/ 0.915	33.16/ 0.963	36.61/ 0.974	37.22/ 0.955	35.20/ 0.956	**37.56/** **0.979**
Rain200L	31.66/ 0.922	38.43/ 0.982	32.20/ 0.929	36.13/ 0.975	37.93/ 0.983	**39.13/** **0.985**	38.99/ 0.983	36.12/ 0.979	38.08/ 0.983

**Table 4:** Quantitative comparison on the Rain1400 dataset. Bolded texts represent the best performance.

	DDN	SIRR	JDDN (Ours)
PSNR	29.91	28.44	**30.98**
SSIM	0.910	0.889	**0.937**

**Table 5:** Quantitative comparison on the SPA-data real dataset. **Bolded** texts represent the best performance.

	DDN	RESCAN	SIRR	SPANet	PReNet	JORDER	JDDN (Ours)
PSNR	34.80	34.73	34.84	35.26	35.00	34.13	**35.31**
SSIM	0.936	0.937	0.936	**0.945**	0.941	0.934	0.937

**Table 6:** Quantitative comparison of ablation experiments. Bolded texts represent the best performance.

	**RMM**	**MCB**	**NLSTM**	**JDDN**
Rain100H	29.14/0.896	29.36/0.900	20.89/0.804	**29.79/0.907**
Rain100L	33.97/0.966	34.26/0.968	19.84/0.880	**37.56/0.979**
Rain200H	29.21/0.899	28.96/0.900	21.21/0.799	**29.43/0.908**
Rain200L	34.33/0.965	33.94/0.967	20.54/0.880	**38.08/0.983**
Rain12	35.96/0.968	35.78/0.967	23.61/0.910	**35.99/0.969**

## Data Availability

All datasets used in this paper are publicly available, RainTrainH, RainTrainL, Rain100H, Rain100L, Rain200H, Rain200L at http://www.icst.pku.edu.cn/struct/Projects/joint_rain_removal.html, (accessed on 13 January 2022), Rain12 at http://yu-li.github.io/paper/li_cvpr16_rain.zip, (accessed on 3 January 2022), Rain1400 and Rain12600 at https://xmu-smartdsp.github.io/, (accessed on 3 January 2022), SPA-data at https://drive.google.com/drive/folders/1eSGgE_I4juiTsz0d81l3Kq3v943UUjiG, (accessed on 3 January 2022).
